# Implementation of Perforated Concentric Ring Walls Considerably Improves Gas-Liquid Mass Transfer of Shaken Bioreactors

**DOI:** 10.3389/fbioe.2022.894295

**Published:** 2022-05-12

**Authors:** Sven Hansen, Andreas Gumprecht, Linda Micheel, Hans-Georg Hennemann, Franziska Enzmann, Wilfried Blümke

**Affiliations:** ^1^ Evonik Operations GmbH, Marl, Germany; ^2^ Evonik Operations GmbH, Hanau-Wolfgang, Germany

**Keywords:** shake flask, oxygen transfer rate, small scale cultivation, RAMOS, bioreactors, *Escherichia coli*

## Abstract

Since their first use in the 1930s, shake flasks have been a widely used bioreactor type for screening and process development due to a number of advantages. However, the limited gas-liquid mass transfer capacities—resulting from practical operation limits regarding shaking frequency and filling volumes—are a major drawback. The common way to increase the gas-liquid mass transfer in shake flasks with the implementation of baffles is generally not recommended as it comes along with several severe disadvantages. Thus, a new design principle for shaken bioreactors that aims for improving the gas-liquid mass transfer without losing the positive characteristics of unbaffled shake flasks is introduced. The flasks consist of cylindrical glass vessels with implemented perforated concentric ring walls. The ring walls improve the gas-liquid mass transfer *via* the formation of additional liquid films on both of its sides, whereas the perforations allow for mixing between the compartments. Sulfite oxidation experiments revealed over 200% higher maximum oxygen transfer capacities (OTR_max_) compared to conventional shake flasks. In batch cultivations of *Escherichia coli* BL21 in mineral media, unlimited growth until glucose depletion and oxygen transfer rates (OTR) of up to 138 mmol/L/h instead of an oxygen limitation at 57 mmol/L/h as in normal shake flasks under comparable conditions could be achieved. Even overflow metabolism could be prevented due to sufficient oxygen supply without the use of unconventional shaking conditions or oxygen enrichment. Therefore, we believe that the new perforated ring flask principle has a high potential to considerably improve biotechnological screening and process development steps.

## Introduction

Seventy-six years after the Erlenmeyer flask was first presented in 1857 (Erlenmeyer, 1860), Kluyver and Perquin (1933) were the first to describe shake flasks for submerse cultivations of microorganisms (Calam, 1969). Since then, shaken bioreactors have become the most widely used type of bioreactors for screening and process development (Büchs, 2001; Klöckner and Büchs, 2012).

In addition to microtiter plates, which are becoming increasingly important, due to their possibilities for high-throughput applications (Hemmerich et al., 2018; Prado and Borges, 2018; Fink et al., 2021), shake flasks are still a widespread and useful type of lab-scale bioreactors. This may be attributed to several advantages they still offer compared to other types of bioreactors:

1) The working volumes of several ml generally still allow for enough possibilities for off-line analytics.
2) With the well-established RAMOS technology, the direct online measurement of oxygen transfer rates (OTR), carbon dioxide transfer rates (CTR), and respiratory quotients (RQ) and thus calibration-free determination of metabolic rates are possible (Anderlei and Büchs, 2001; Anderlei et al., 2004). Additionally, defined volumetric gas flow rates as in fermenters can be applied.
3) Fed-batch mode for multiple components can be implemented in a very cost-effective way (Jeude et al., 2006; Panula-Perälä et al., 2008; Krause et al., 2010; Bähr et al., 2012; Krause et al., 2016; Philip et al., 2017; Habicher et al., 2019; Keil et al., 2019; Müller et al., 2019; Teworte et al., 2021). This even includes pH control and, thus, by reducing buffer concentrations, similar osmotic conditions as commonly used in stirred tank bioreactors can be applied (Scheidle et al., 2011; Philip et al., 2018).
4) The extensive characterization with respect to engineering parameters such as power input (Büchs et al., 2000a; Büchs et al., 2000b; Büchs et al., 2001; Peter et al., 2006a; Peter et al., 2006b), mixing times (Tan et al., 2011), hydrodynamics (Büchs et al., 2001; Peter et al., 2004; Büchs et al., 2007; Tianzhong et al., 2010; Liu et al., 2016; Palacios-Morales et al., 2016; Azizan and Büchs, 2017; Azizan et al., 2019), and gas-liquid mass transfer (Van Suijdam et al., 1978; Henzler and Schedel, 1991; Maier and Büchs, 2001; Maier et al., 2004; Giese et al., 2014) enables good and reliable experimental designs.
5) Due to surface aeration, complicating phenomena that usually come along with bubble aeration are avoided. Therefore, in contrast to stirred tank reactors or baffled shaken bioreactors, the gas-liquid mass transfer can be determined and controlled much easier and in a more reliable manner even with varying and changing media compositions (Büchs, 2001; Maier et al., 2004).
6) The good computability of the gas-liquid mass transfer and the extensive characterization also allows for the synchronization and parallelization with other shaken bioreactors like microtiter plates. Thus, these parallelized reactors can be regarded as one cultivation that can massively increase the data insight by coupling different measurement technologies and enabling noninvasive sampling (Wewetzer et al., 2015).

All these points not only make the shake flask a highly valuable screening tool but also a very useful and cost-efficient reactor type for bioprocess development.

However, besides all these advantages, shake flasks still exhibit a major drawback compared to stirred tank bioreactors, namely the significantly lower gas-liquid mass transfer capacities (McDaniel et al., 1965; Van Suijdam et al., 1978). Whereas, in stirred tank reactors OTRs of 100–150 mmol/L/h can be achieved on production relevant scale (Van’t Ried, 1979; Yawalkar et al., 2008; Garcia-Ochoa and Gomez, 2008), in shake flasks hardly much higher maximum oxygen transfer capacities (OTR_max_) than 60 mmol/L/h can be achieved in practice even under advantageous conditions (Freyer et al., 2004; Meier et al., 2016). However, in aerobic cultivations the avoidance of oxygen limitations is usually imperative and, therefore, requires special attention in the experimental design (McDaniel et al., 1965; Losen et al., 2004).

A common way to increase the gas-liquid mass transfer in shake flasks is the introduction of baffles. The positive effect with respect to oxygen supply has already been described decades ago (Smith and Johnson, 1954; Gaden, 1962; McDaniel et al., 1965). However, the use of baffles also comes along with several severe disadvantages: due to the chaotic splashing of the liquid, the hydrodynamics become much more complex (Büchs, 2001; Li et al., 2013). The advantage of the good computability of the gas-liquid mass transfer is thus abandoned. In addition, the reproducibility of experiments also suffers, because the baffles themselves are generally not produced in a reproducible manner, due to the manufacturing properties of glass and the manual implementation of the baffles (McDaniel et al., 1965; Freedman, 1969; Delgado et al., 1989; Büchs, 2001). However, it must be admitted, that at least reproducible geometries can be achieved when using plastic flasks (Brodsky and Cronin, 2006; Ukkonen et al., 2011; Running and Bansal, 2016). Nonetheless, and even more severe, baffles can massively increase the risks of achieving out-of-phase phenomena, excessive foaming, and splashing of liquid up to the sterile barrier. These phenomena can even lead to a decreasing gas-liquid mass transfer (McDaniel et al., 1965; Henzler and Schedel, 1991; Büchs, 2001; Büchs et al., 2001; Running and Bansal, 2016). Therefore, unless high energy dissipation is necessary (Peter et al., 2006b), the use of baffled shake flasks is usually discouraged.

To circumvent these drawbacks, further approaches to increase the gas-liquid mass transfer in shaken bioreactors have been published. The enrichment of air with oxygen increases the OTR_max_ by increasing the driving concentration gradient of oxygen and can avoid oxygen limitations even in heavily breathing cultures (Losen et al., 2004). Due to safety considerations, however, this method is very elaborate. Additionally, it can only increase the mass transfer for the enriched compound but would, e.g., neglect the removal of carbon dioxide. Another approach is the attachment of a helical track at the inner wall of a cylindrical vessel to increase the gas-liquid interface area. With this concept, a clear improvement in oxygen transfer could be shown for a wide range of vessel sizes (Zhang et al., 2008). Recently, Zhu et al. (2019) presented an orbitally shaken bioreactor featuring a hollow cylinder wall. However, although achieving improved mixing behavior the authors observed a negative influence on the volumetric mass transfer coefficient (k_L_a). In contrast, Wang et al. (2021) could improve the k_L_a without increasing the shear stress by implementing a vaulted bump into a shaken bioreactor.

In this study, a new design principle for increasing the gas-liquid mass transfer in shake flasks is proposed. The presented flasks contain internal, concentric ring walls with perforations to allow the mixing of the liquids on both sides of the ring wall. The design intention of this perforated ring flask is to significantly increase the liquid film area during shaking. This enables higher gas-liquid mass transfers even under practical operating conditions without losing the positive characteristics of unbaffled shake flasks as aforementioned.

## Materials and Methods

### Respiration Activity Monitoring System

For the determination of OTRs in the shaken flasks the respiration activity monitoring system (RAMOS) was used in several experiments of the study. This system allows the noninvasive determination of respiration rates in modified shake flasks. The measuring principle as introduced by Anderlei and Büchs (2001) and Anderlei et al. (2004) was applied. The set-up of a RAMOS device is illustrated in the Supplementary Material.

### Flasks

**FIGURE 1:**
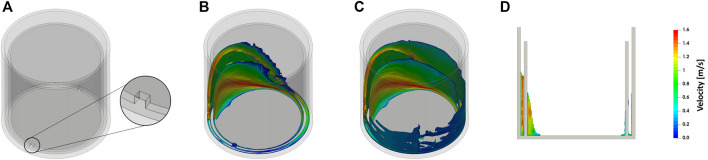
Design and working principle of the perforated ring flask. **(A)** Illustration of an empty perforated ring flask. **(B)** Illustration of the bulk liquid within a perforated ring flask. **(C)** Illustration of the bulk liquid and the liquid film within a perforated ring flask. **(D)** Side view of a perforated ring flask with liquid. The images are created based on a CFD simulation as described in the *Materials and Methods* section. Simulation conditions: inner vessel diameter d_i_ = 80 mm, inner ring diameter d_ring,i_ = 70.6 mm, ring wall thickness d_wall_ = 2.2 mm, and 2 perforations with 3 mm × 3 mm size; filling volume V_L_ = 10 ml; shaking diameter d_0_ = 50 mm; shaking frequency *n* = 300 rpm.

Figure 1A shows the general concept of the newly proposed flask design. The flask basically consists of a cylindrical vessel. Inside this vessel, a concentric ring wall is attached to the flat flask bottom and, thereby, divides the flask into two compartments. The ring wall is perforated twice directly adjacent to the flask bottom. The purpose of these perforations is to allow the liquid to pass the ring wall from one compartment to the other and, thereby, prevent the formation of two separate populations during cultivation. Based on this principle several sets of prototypes have been manufactured.

The first set of prototypes consists of a cylindrical glass vessel with a flat bottom and two cylindrical glass vessels with flat bottoms and inner cylindrical, concentric ring walls. Both the vessels and the inner ring walls have a height of h = 120 mm. The vessels have an inner diameter of d_i_ = 55.6 mm and a wall thickness of d_wall_ = 2.2 mm. The cylindrical ring wall of one vessel has an inner diameter of d_ring,i_ = 35.2 mm and wall thickness of d_wall_ = 1.4 mm, resulting in a gap size between the ring wall and the vessel wall of d_gap_ = 8.8 mm. The cylindrical ring wall of the other vessel had an inner diameter of d_ring,i_ = 40.8 mm and wall thickness of d_wall_ = 1.6 mm, resulting in a gap size between the ring wall and the vessel wall of d_gap_ = 5.6 mm. Both cylindrical ring walls have four equidistant perforations of roughly 2 mm × 2 mm size directly adjacent to the bottom.

The second set of prototypes consists of cylindrical glass tubes that have been integrated into a conventional RAMOS flask by a glassblower to allow OTR measurements with a RAMOS device. A reference flask includes only the vessel wall with an inner diameter of d_i_ = 55.6 mm, a wall thickness of d_wall_ = 2.2 mm, and a height of h = 50 mm. The other flasks of this set additionally include an inner cylindrical ring with an inner diameter of d_ring,i_ = 40.8 mm, a wall thickness of d_wall_ = 1.6 mm resulting in a gap size between the ring wall and the vessel wall of d_gap_ = 5.6 mm, and a height of h_ring_ = 50 mm. These cylindrical rings have different perforations directly adjacent to the bottom. In the case of more than one perforation, these perforations are spaced equidistantly. The following perforations have been manufactured: 4 perforations with 2 mm × 2 mm, 1 perforation with 2 mm × 4 mm, 2 perforations with 2 mm × 2 mm, 2 perforations with 3 mm × 3 mm, and 2 perforations with 2 mm × 4 mm.

The third set of prototypes consists of cylindrical glass vessels with flat bottoms and inner cylindrical, concentric ring walls. The vertical parts of the vessel walls have a height of h = 80 mm. The inner ring walls have a height of h_ring_ = 65 mm. The vessels have an inner diameter of d_i_ = 80 mm and a wall thickness of d_wall_ = 2.5 mm. The ring walls have an inner diameter of d_ring,i_ = 70.6 mm and a wall thickness of d_wall_ = 2.2 mm, resulting in a gap size between the ring wall and the vessel wall of d_gap_ = 2.5 mm. Two equidistantly spaced perforations with 3 mm × 3 mm have been integrated into the ring walls directly adjacent to the bottom. Additionally, this flask version features a top of a conventional RAMOS flask including ports for the oxygen sensor, the gas inlet, and the gas outlet.

All prototypes have been handcrafted by a glassblower with borosilicate glass 3.3. Drawings of all prototype versions are added in the Supplementary Section.

### Sulfite Oxidation Experiments

Sulfite oxidation experiments have been performed as described by Hermann et al. (2001) with a sulfite solution that consists of 0.5 mol/L Na_2_SO_3_ (Sigma-Aldrich, Steinheim, Germany), 0.012 mol/L Na_2_HPO_4_/NaH_2_PO_4_ (Sigma-Aldrich, Steinheim, Germany), and 10^−7^ mol/L CoSO_4_ (Sigma-Aldrich, Steinheim, Germany). The pH value was adjusted to 8 with sulfuric acid (Bernd Kraft, Duisburg, Germany). In experiments without RAMOS additionally 2.4 × 10^−5^ mol/L bromothymol blue (Sigma-Aldrich, Steinheim, Germany) was included in the solution.

In the sulfite oxidation experiments without RAMOS, the flasks (first prototypes) have been covered with AirOTop™ Enhanced Flask Seals for Ultra Yield^®^ 2.5 L Flask (Thomson Instrument Company, Oceanside, United States) to avoid evaporation losses. A shaking machine (LSR-V-12.5, Kuehner AG, Birsfelden, Switzerland) with a shaking diameter of d_0_ = 25 mm has been used at a temperature of T = 21°C. Filling volumes of the flasks and shaking frequencies varied and are specified in the discussion section. The color change has been detected with Samsung Galaxy A3 (2016) (Samsung, Suwong, South Korea) and the app “Simple Interval Camera” (esukeapp).

The sulfite oxidation experiments in the RAMOS device have been performed on a shaking machine (ISF-1-W, Kuehner AG, Birsfelden, Switzerland) with a shaking diameter of d_0_ = 50 mm at a temperature of T = 37°C. Filling volumes of the flasks and shaking frequencies varied and are specified in the discussion section.

### Microorganisms

*Escherichia coli* BL21 (DE3) was used as a model organism in this study. The microorganisms were maintained at −80°C in Lysogeny broth (LB) medium with 15% glycerol content.

### Media

LB medium was used for the preparation of cryocultures and the cultivation of precultures. The medium consists of 5 g/L yeast extract (powdered, Carl Roth, Karlsruhe, Germany), 10 g/L tryptone (pancreatic digest of casein, Carl Roth, Karlsruhe, Germany), and 10 g/L NaCl (Sigma-Aldrich, Steinheim, Germany).

Main cultures of *E. coli* BL21 were cultivated in a modified Wilms & Reuss synthetic medium (henceforth referred to as Wilms-MOPS medium) (Wilms et al., 2001; Scheidle et al., 2007; Hansen et al., 2012). This medium consists of varying concentrations of glucose (further specified in the corresponding section of this article), 5 g/L (NH_4_)_2_SO_4_ (Carl Roth, Karlsruhe, Germany), 0.5 g/L NH_4_Cl (Carl Roth, Karlsruhe, Germany), 3 g/L K_2_HPO_4_ (Carl Roth, Karlsruhe, Germany), 2 g/L Na_2_SO_4_ (Merck, Darmstadt, Germany), 0.5 g/L MgSO_4_·7H_2_O (Th. Geyer, Renningen, Germany), 41.85 g/L (0.2 M) 3-(N-morpholino)-propanesulfonic acid (MOPS) (Carl Roth, Karlsruhe, Germany), 0.01 g/L thiamine hydrochloride (Carl Roth, Karlsruhe, Germany), 1 ml/L trace element solution [0.54 g/L ZnSO_4_·7H_2_O (Merck, Darmstadt, Germany), 0.48 g/L CuSO_4_·5H_2_O (Merck, Darmstadt, Germany), 0.3 g/L MnSO_4_·H_2_O (Riedel de Haen, Seelze, Germany), 0.54 g/L CoCl_2_·6H_2_O (Merck, Darmstadt, Germany), 41.76 g/L FeCl_3_·6H_2_O (Carl Roth, Karlsruhe, Germany), 1.98 g/L CaCl_2_·2H_2_O (Merck, Darmstadt, Germany), and 33.39 g/L Na_2_EDTA (Titriplex III) (Carl Roth, Karlsruhe, Germany)].

### Cultivation Conditions

Precultures of *E. coli* were inoculated with 100 μl cryoculture and were cultivated in 250 ml baffled Erlenmeyer flasks at a temperature of T = 37°C on a shaking machine (InforsHT-Mulitron, Infors AG, Bottmingen, Switzerland) with a shaking diameter of d_0_ = 25 mm, a shaking frequency of *n* = 200 rpm, and a filling volume of V_L_ = 25 ml.

Main cultures of *E. coli* were inoculated with broth from the precultures resulting in an optical density at 600 nm (OD_600_) of 0.1. The main cultures were cultivated in conventional RAMOS flasks and the aforementioned prototype vessels using a RAMOS device from HiTec Zang (Herzogenrath, Germany) to measure OTRs. The RAMOS device was placed on a shaking machine (ISF-1-W, Kuehner AG, Birsfelden, Switzerland) with a shaking diameter of d_0_ = 50 mm. The applied shaking frequencies varied among the experiments and are specified in the corresponding section of this study. Cultivation temperatures of T = 37°C had been set.

### Software

The CFD simulations have been performed by the open-source software OpenFOAM v8. An incompressible two-phase solver, with the underlying volume of the fluid method, has been used. The two-phase system looked at, is water and air at a temperature of 32°C. For illustrating the film on the vessel walls, a contact angle of 20° has been set.

## Results and Discussion

### Principle of the Perforated Ring Flask

As shown by Maier and Büchs (2001), in orbitally shaken glass flasks the liquid film that forms on the hydrophilic surface is the main contributor to the gas-liquid mass transfer. Whereas the implementation of baffles focuses on the smaller contributor by trying to rip the bulk liquid, this study focuses on increasing the liquid film area without increasing the flask diameter and without impacting the laminar character of the hydrodynamics. This intensification of film area is supposed to considerably increase the gas-liquid mass transfer and shall be realized with the implementation of concentric, perforated ring walls into the flasks.

For an illustration of the new flask design and the working principle, a CFD model was created. Figure 1 shows both an exemplary design of the perforated ring flask and the liquid hydrodynamics resulting from this CFD simulation. Figure 1B gives an impression of the form of the bulk liquid in the perforated ring flask. The formed liquid sickle looks very similar to the one that can be seen in other cylindrical vessels without an internal ring wall (Klöckner et al., 2014; Zhu et al., 2017a; Zhu et al., 2017b). Figure 1C additionally illustrates the liquid films that form on the surfaces of the walls. The simulation confirms that all three walls are wetted with a liquid film and, consequently, three liquid films instead of only one are formed. Figure 1D shows a side view into the flask and additionally confirms these statements. On the left-hand side of this illustration, the bulk liquid can be seen, whereas on the right-hand side the thin liquid films on all three walls are visible. Therefore, an increase in the total film area compared to a normal cylindrical vessel can be reasonably expected. Consequential, this increase in the total film area is assumed to considerably increase the gas-liquid mass transfer.

As the CFD simulation was only performed for the sake of visualization and was not validated with experimental data, no deeper, quantitative conclusions are drawn from these calculations. Therefore, experimental investigations with prototypes made of glass have been performed.

### Validation of Principle and Influence of Ring Wall Diameter

To validate the general hypothesis, that the perforated concentric ring walls can improve the gas-liquid mass transfer of shaken bioreactors, three different glass prototypes have been manufactured. These prototypes consist of cylindrical vessels, whereas two of them contained perforated concentric ring walls. OTR_max_ in these prototypes has been determined using the sulfite oxidation method. Details about the design of the prototypes and the sulfite oxidation method are described in the *Materials and Methods* section.

**FIGURE 2:**
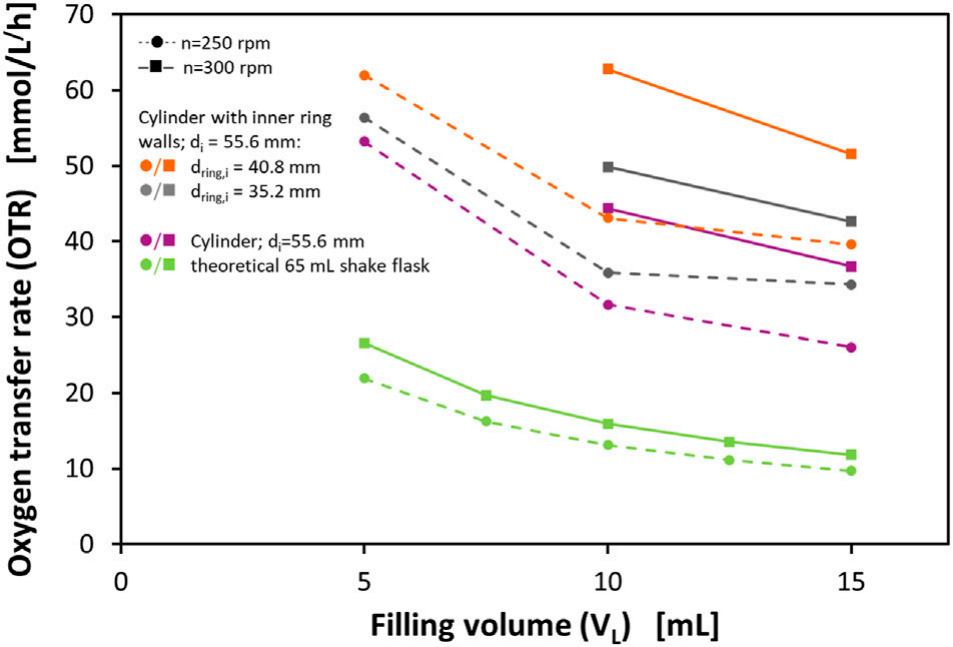
Maximum oxygen transfer capacities (OTR_max_) of a cylindrical vessel and two different perforated ring flasks in a 0.5 M sulfite oxidation system and calculated OTR_max_ levels for a theoretical 65 ml shake flask in 0.5 M sulfite oxidation system according to Meier et al. (2016). The cylindrical vessel and the perforated ring flasks have an inner diameter of d_i_ = 55.6 mm, the perforated ring flasks have ring diameters of d_ring,i_ = 35.2 mm, and d_ring,i_ = 40.8 mm, respectively, wall thicknesses of the rings of d_wall_ = 1.4 mm and d_wall_ = 1.6 mm, respectively, and 4 equidistant perforations directly adjacent to the bottom with a size of 2 mm × 2 mm; Experimental conditions: 0.5 M Na_2_SO_3_, 0.012 M phosphate buffer, 10^−7^ M CoSO_4_, and 2.4 × 10^−5^ M bromothymol blue; initial pH = 8; temperature T = 21°C; shaking diameter d_0_ = 25 mm.

Figure 2 shows the determined OTR_max_ at different filling volumes and shaking frequencies. Additionally, the figure shows the corresponding OTR_max_ of a theoretical 65 ml shake flask according to the correlation of Meier et al. (2016). With this correlation, the OTR_max_ in normal shake flasks (Erlenmeyer flasks) can be predicted based on the maximum flask diameter d, the shaking frequency n, the shaking diameter d_0_, the liquid filling volume V_L,_ and the osmolarity of the medium as the only medium parameter. Although 65 ml is not a commonly available nominal volume of Erlenmeyer flasks, this size would correspond to the volume of an Erlenmeyer flask with the same maximum inner diameter as the examined vessels. Therefore, the prototype vessels can fairly be compared to the theoretical 65 ml shake flask as they would need the same amount of space on a shaker tray.

The measured OTR_max_ in the perforated ring flask prototypes decreases with increasing filling volumes and increases with increasing shaking frequencies. These observations are very well in agreement with the findings of other authors for shake flasks (McDaniel et al., 1965; Van Suijdam et al., 1978; Maier and Büchs, 2001; Maier et al., 2004). For all examined experimental conditions, the cylindrical vessel without internal ring walls already shows remarkably higher OTR_max_ than the theoretical 65 ml shake flask. This can be explained by the different geometries of the vessel wall. In the mainly conical Erlenmeyer flask, the inner diameter is at its maximum in only one horizontal slice of the flask. In contrast, in a cylinder, the maximum flask diameter applies to all horizontal slices of the vessel. That means that in cylindrical vessels almost all parts of the liquid at the glass wall experience a higher circumferential velocity than in a normal shake flask. Additionally, the liquid can move up higher on the vessel wall and, therefore, a bigger film area can develop resulting in a higher OTR. This observation has already been made by Akgün et al. (2008) in a continuous parallel shaken bioreactor system.

On top of this effect, the perforated ring flasks show a further improvement in the gas-liquid mass transfer. This can be explained by the larger total film area that can be realized by the addition of the ring walls. Two more liquid films contribute to this total film area when one perforated ring wall is implemented, namely both at the inner and the outer side of the ring wall (see Figure 1). As already pointed out, the liquid film on the glass wall has a significant impact on the oxygen transfer. The increase of the OTR_max_ is higher for the vessel with a larger diameter of the ring wall. This observation is valid for all examined filling volumes and shaking frequencies. Again, this can be explained by the size of the liquid film area. With a larger ring wall diameter, the liquid can spread over a wider range of the glass surface and, therefore, a bigger total film area can be formed that contributes to a higher gas-liquid mass transfer.

In addition to the presented data, another observation made during the experiments is worth to be mentioned, namely that virtually no splashing of liquid can be observed during the shaking in any of the flasks. This indicates that the bulk liquid is not significantly disrupted as in baffled flasks and that the improved gas-liquid mass transfer indeed mainly results from an increase in the liquid film area. Consequently, a higher oxygen transfer can be realized without the need to accept the disadvantages that come along with baffles.

The sulfite oxidation experiment with the first set of prototypes showed that a clear improvement of the gas-liquid mass transfer can be achieved with both vertical vessel walls and additionally perforated concentric ring walls, whereas ring walls with a larger diameter show a bigger improvement. However, with the sulfite oxidation experiment, no conclusions about the local gas-liquid mass transfer and its impact on the bulk homogeneity can be drawn as only the total amount of oxygen consumed is determined. If one of both compartments would exhibit a lower oxygen supply in combination with poor mixing between the two compartments, this could still have a negative impact on biological cultivations. Therefore, the newly proposed flask design shall be evaluated in a real cultivation.

### Transferability to *E. coli* Cultivations and Influence of Perforation Design

For this reason, new flasks have been manufactured. In contrast to the first generation of prototypes used for sulfite oxidation, the new versions were integrated into usual RAMOS flasks. Here, the RAMOS flasks serve as supporting structures for the integration of the perforated ring flasks into a RAMOS device, whereas the cylindrical vessel wall still is the actual outer reactor wall. In this manner, the cylindrical vessel from the previous experiment has been manufactured. Additionally, five perforated ring flasks with an inner ring wall diameter of d_ring,i_ = 40.8 mm have been manufactured. These five-ring flasks are perforated in different ways to examine the influence of the shape and the size of the perforations. A detailed description of the flask designs can be found in the *Materials and Methods* section.

**FIGURE 3:**
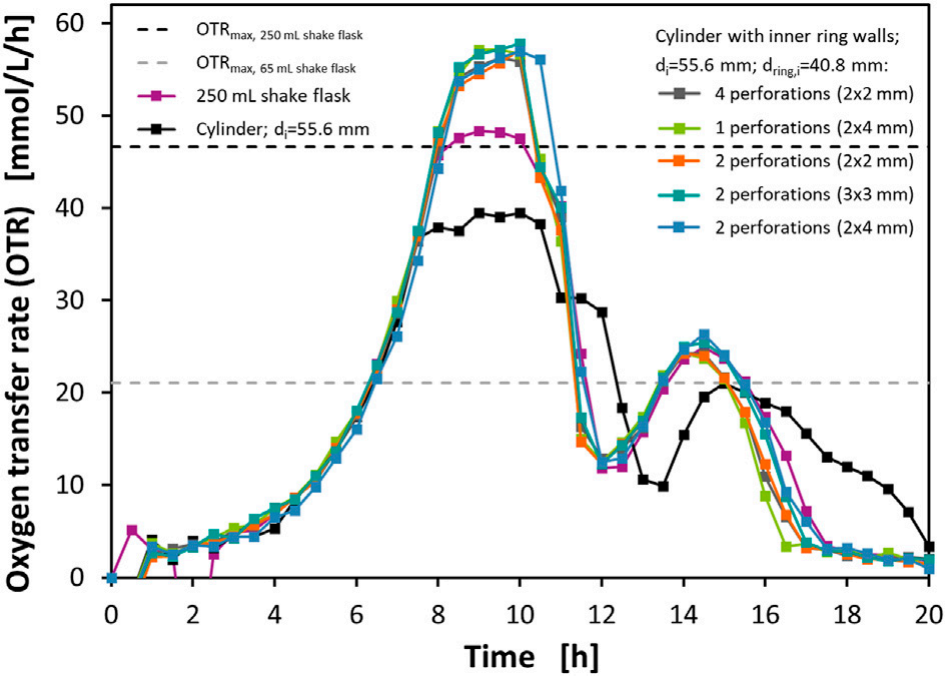
Oxygen transfer rates (OTR) from cultivations of *E. coli* BL21 in mineral medium in perforated ring flasks with inner vessel diameter d_i_ = 55.6 mm, inner ring diameter d_ring,i_ = 40.8 mm, and wall thickness d_wall_ = 1.6 mm. The full lines with data points show OTRs from different flasks measured with RAMOS. The dashed horizontal lines show the predicted OTR_max_ levels for a 250 ml shake flask and a theoretical 65 ml shake flask according to Meier et al. (2016). Experimental conditions: Wilms-MOPS medium with 23 g/L glucose; temperature T = 37°C; filling volume V_L_ = 10 ml; shaking diameter d_0_ = 50 mm; shaking frequency *n* = 250 rpm; initial optical density OD_600_ = 0.1.

With these flasks, cultivations of *E. coli* BL21 in Wilms-MOPS mineral medium with 23 g/L glucose were performed using a RAMOS device to measure the OTRs. As a reference, additionally, the same cultivation was performed in a normal RAMOS flask representing a 250 ml shake flask (Anderlei and Büchs, 2001; Anderlei et al., 2004). Similar cultivation systems with normal RAMOS flasks and different variations in media composition, cultivations conditions, and technical set-ups have already been described by various authors (Scheidle et al., 2007; Hansen et al., 2012; Philip et al., 2017; Philip et al., 2018). Therefore, the cultivation of *E. coli* BL21 in Wilms-MOPS mineral medium serves as a well-known model system. The cultivations were performed at T = 37°C, with a filling volume of V_L_ = 10 ml, a shaking diameter of d_0_ = 50 mm, and a shaking frequency of *n* = 250 rpm. Figure 3 shows the measured OTRs of these experiments. Additionally, the calculated OTR_max_ levels according to Meier et al. (2016) for a 250 ml shake flask and a theoretical 65 ml shake flask with the same medium and at the same operating conditions are shown.

The curve representing the normal RAMOS flask shows a course that is well known from the mentioned literature. After an exponential increase of the OTR up to 8 h the curve flattens to a plateau of around 48 mmol/L/h. This plateau is the result of an oxygen limitation and, therefore, marks the OTR_max_ of the normal RAMOS flask in this medium at the given operating conditions. This is also in good agreement with the correlation found by Meier et al. (2016) that predicts the OTR_max_ at this level. After 10 h the curve declines again which is due to the depletion of glucose. In contrast to the results of other authors (Scheidle et al., 2007; Hansen et al., 2012; Philip et al., 2017; Philip et al., 2018), the drop in OTR is not that harsh at this point. Instead, a slight shoulder can be seen in this curve. This is probably due to small amounts of glycerol that were brought into the main medium *via* the preculture. After this short effect, the OTR declines to a level of around 12 mmol/L/h at 12 h before a second peak forms with its maximum of 25 mmol/L/h at 15 h. This second, diauxic peak results from the metabolization of acetate that has been formed during the metabolization of glucose in the first peak due to overflow metabolism as described in detail in the literature (Scheidle et al., 2007; Hansen et al., 2012; Philip et al., 2017; Philip et al., 2018). After 15 h the curve declines again, and the cultivation comes to an end.

The cylindrical vessel is initially identical to the curve of the normal RAMOS flask. After 7.5 h, however, a plateau of around 39 mmol/L/h is reached. Again, this plateau marks an oxygen limitation and, therefore, indicates the OTR_max_ of this flask at the given operating conditions. The plateau lasts until 10.5 h, then again a shoulder, most probably caused by the glycerol from the preculture appears followed by a diauxic acetate peak starting after 13.5 h. The focus of this experiment, however, is on the height of the OTR_max_ level. Although the achieved value of 39 mmol/L/h is still below the OTR_max_ of the normal RAMOS flask it is clearly above the level of 21 mmol/L/h that would be expected from the theoretical 65 ml shake flask. The lower OTR_max_ of the 65 ml shake flask compared to the 250 ml shake flask results from the smaller maximum flask diameter that decreases the circumferential velocity of the liquid and the potential liquid film area. Therefore, the theoretical 65 ml shake flask is a fair comparison to the prototypes, as it has the same maximum flask diameter. The results show again that the vertical geometry of the wall leads to a superior gas-liquid mass transfer compared to the conical shape of an Erlenmeyer flask. This observation very well agrees with the one made in the sulfite oxidation experiment illustrated in Figure 2 and with the findings of Akgün et al. (2008).

All the other five curves in Figure 3, representing the flasks with the perforated concentric ring walls, are nearly identical. The first phase of the cultivation with the exponential growth is even identical to the OTR curves from the normal RAMOS flask and the reference flask but in this case the OTRs rise higher than in both other flasks and, after 8.5 h, reach plateaus of around 56 mmol/L/h. Although these plateaus are not completely horizontal to the time axis, they can be attributed to oxygen limitations. This phenomenon could already be seen in an earlier study where it could be clearly attributed to an oxygen limitation with the help of a more advanced data analysis method (Hansen et al., 2012). The reasons for these increasing plateaus are most probably changes in the oxygen solubility and the diffusion coefficient due to a changing media composition. After the depletion of the glucose again a decrease with a shoulder and subsequently following acetate peak can be seen.

Again, the focus of this experiment is on the maximum OTRs that could be achieved. These OTR_max_ in the perforated ring flasks are not only higher than in the reference flask but also outperform the normal RAMOS flask although exhibiting a smaller maximum flask diameter. According to the correlation of Meier et al. (2016), an OTR of 56 mmol/L/h in 250 ml shake flasks at elsewise the same conditions could only be achieved with a shaking frequency of at least *n* = 300 rpm. For a theoretical 65 ml shake flask, which serves as a fair comparison to the evaluated perforated ring flasks, even a shaking frequency of 600 rpm is predicted to achieve a comparable OTR_max_. Although this shaking frequency is outside of the experimentally evaluated range, it shows that shaking frequencies far above the practically applicable range would be needed to achieve comparable OTRs. In total, the outcome of this experiment shows that the perforated concentric ring walls have a highly positive impact on the gas-liquid mass transfer which is not restricted to a chemical model system but can also be transferred to real cultivations. Additionally, the identical levels of the OTR_max_ and the nearly identical shapes of the curves indicate that the size and geometries of the evaluated perforations have no severe impact on the hydrodynamics. At least the gas-liquid mass transfer characteristics are the same in all the examined perforated ring flasks.

### Characterization of Maximum Oxygen Transfer Capacities With 0.5 M Sulfite Oxidation System

**FIGURE 4:**
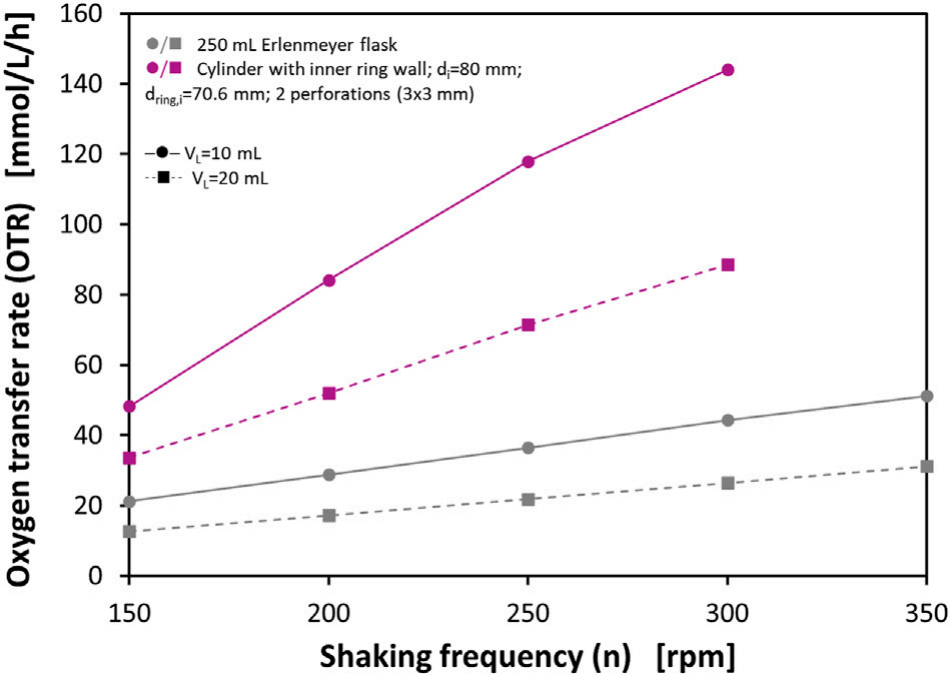
Maximum oxygen transfer capacities (OTR_max_) of a perforated ring flask with inner vessel diameter d_i_ = 80 mm, inner ring diameter d_ring,i_ = 70.6 mm, wall thickness d_wall_ = 2.2 mm, and 2 perforations of 3 mm × 3 mm size in 0.5 M sulfite system. The data show both measured OTR_max_ with RAMOS of cylindrical vessels and perforated ring flasks and calculated OTR_max_ levels for a 250 ml shake flask according to Meier et al. (2016). Experimental conditions: 0.5 M Na_2_SO_3_, 0.012 M phosphate buffer, 10^−7^ M CoSO_4_; initial pH = 8; temperature T = 37°C; shaking diameter d_0_ = 50 mm.

Probably the most frequently used shake flask is the 250 ml Erlenmeyer flask. This flask with a maximum outer diameter of approx. 85 mm is also most frequently described in the literature when used with RAMOS. Since the flask diameter also has an influence on the OTR_max_ (Henzler and Schedel, 1991; Maier and Büchs, 2001; Meier et al., 2016), further perforated ring flasks with the same maximum flask diameter as the 250 ml shake flask have been manufactured. Consequently, the inner diameter of the cylindrical wall is d_i_ = 80 mm. The inner ring wall has an inner diameter of d_ring,i_ = 70.6 mm, and a wall thickness of d_wall_ = 2.2 mm. Each ring wall has two equidistantly spaced perforations directly adjacent to the bottom with a size of 3 mm × 3 mm. These flasks were characterized with respect to OTR_max_ with the 0.5 M sulfite oxidation system and a RAMOS device using filling volumes of V_L_ = 10 ml and V_L_ = 20 ml, respectively. Figure 4 shows the measured OTRs at different shaking frequencies of *n* = 150–300 rpm. Furthermore, literature data for OTR_max_ in 250 ml shake flasks under analogous conditions up to *n* = 350 rpm are shown. These values are calculated based on the correlation of Meier et al. (2016).

For the 250 ml shake flask the curves show OTR_max_ between 12.7 and 51.2 mmol/L/h under the given operating conditions. Higher shaking frequencies and lower filling volumes correlate with higher OTR_max_. The flasks with the perforated ring walls show basically the same trends with respect to shaking frequency and filling volume, but they lead to considerably higher OTRs. For example, at *n* = 150 rpm higher OTRs are measured than in the normal shake flask at 350 rpm with the same filling volumes. At 300 rpm, the highest OTRs can be achieved. For V_L_ = 20 ml the OTR is 88.6 mmol/L/h and for V_L_ = 10 ml the OTR even reaches a level of 144 mmol/L/h. In both cases, this is over 200% higher than the corresponding values from the said correlation. In particular, the measured OTR at V_L_ = 10 ml at *n* = 300 rpm is comparable to OTRs that can usually only be achieved in stirred tank bioreactors. As already pointed out, these much higher OTRs in the perforated ring flask can be explained by the formation of two additional film layers in the flask, which considerably contribute to the gas-liquid mass transfer.

In contrast to the experiments that have been subjected to the correlation of Meier et al. (2016), the experiments in this study could only be carried out up to a shaking frequency of *n* = 300 rpm, as this was the upper limit of the available shaker. Since many commercially available shakers have their maximum at *n* = 300 rpm or even lower, increasing the OTR_max_ beyond this operating point is usually not an option. This once again underlines the strength of the presented technology, which makes it possible to achieve very high OTR_max_ at practical operation conditions on widely commercially available shakers.

### Case Study: *E. coli* Cultivations in Perforated Ring Flasks

**FIGURE 5:**
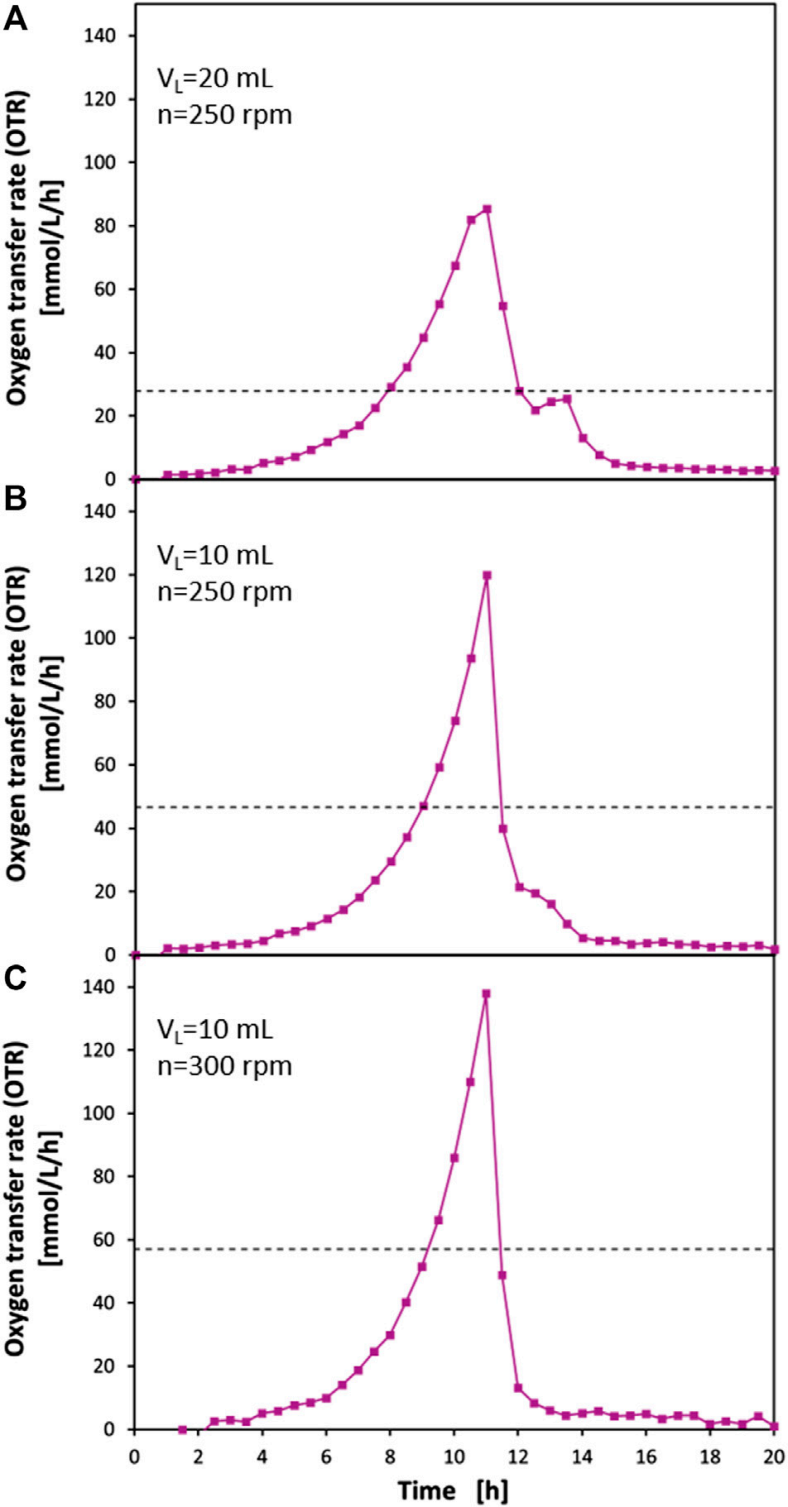
Oxygen transfer rates (OTR) from cultivations of *E. coli* BL21 in mineral medium in perforated ring flasks with inner vessel diameter d_i_ = 80 mm, inner ring diameter d_ring,i_ = 70.6 mm, wall thickness d_wall_ = 2.2 mm, and 2 perforations of 3 mm × 3 mm size. The full lines with data points show OTRs measured with RAMOS. **(A)** filling volume V_L_ = 20 ml; shaking frequency *n* = 250 rpm. **(B)** V_L_ = 10 ml; *n* = 250 rpm. **(C)** V_L_ = 10 ml; *n* = 300 rpm. **(C)** is shifted by 1.5 h. The dashed horizontal lines represent the predicted OTR_max_ levels for 250 ml shake flasks at the corresponding conditions according to Meier et al. (2016). Experimental conditions: Wilms-MOPS medium with 23 g/L glucose; temperature T = 37°C; shaking diameter d_0_ = 50 mm; initial optical density OD_600_ = 0.1.

To demonstrate the advantages of the presented perforated ring flasks with an inner flask diameter of d_i_ = 80 mm in practical applications, exemplary cultivations of *E. coli* BL21 in Wilms-MOPS medium with 23 g/L glucose have been performed in these flasks. Figure 5 shows the results of these cultivations under different operating conditions. Additionally, the OTR_max_ of the normal 250 ml shake flasks is shown, which is calculated with the correlation of Meier et al. (2016).

Figure 5A shows a cultivation carried out with a filling volume of V_L_ = 20 ml and a shaking frequency of *n* = 250 rpm. The dashed line at approximately 28 mmol/L/h indicates the OTR_max_ of a normal 250 ml shake flask under these operating conditions. This means that, as soon as this value is reached, the oxygen uptake of the microorganisms would be limited by the gas-liquid mass transfer and an OTR plateau would form at this level until the glucose is depleted or another limitation or inhibition would occur. The OTR curve of the cultivation in the perforated ring flask, however, shows exponential growth up to 10.5 h after the start of the cultivation and clearly exceeds the value of 28 mmol/L/h. After 11 h, the highest OTR value of approx. 85 mmol/L/h is reached. At this point, also a bending of the curve towards a plateau can be seen, which strongly indicates the presence of an oxygen limitation. The OTR at this point is approximately 19% higher than the OTR_max_ measured in the sulfite oxidation system under the same operating conditions as presented in Figure 4 where an OTR value of 71.5 mmol/L/h was measured. The higher OTR value of the biological system can be explained by the lower salt content of the medium compared to the sulfite oxidation system, which leads to a higher oxygen solubility (Rischbieter and Schumpe, 1996; Weisenberger and Schumpe, 1996). After 11 h, there is a drop in the OTR curve, which can be explained by a depletion of glucose. Afterward, between 12.5 and 14 h, a diauxic peak can be seen. Here, acetate is metabolized, which was previously built up due to the overflow metabolism of *E. coli*. This distinct acetate peak is another strong indication of an oxygen limitation in the last phase of glucose consumption. Overall, the OTR curve is very similar to those described in the literature for operating conditions of V_L_ = 10 ml and *n* = 350 rpm (Scheidle et al., 2007; Hansen et al., 2012; Philip et al., 2018). In these cases, an oxygen limitation at an OTR level of approx. 60 mmol/L/h can be seen. The subsequent diauxic peak has a similar size to that seen in this experiment and was clearly identified as an acetate peak by offline measurements. Nonetheless, it is noteworthy that in the perforated ring flask a higher OTR_max_ can be achieved than in normal shake flasks under more favorable shaking conditions.

Figure 5B shows the same experimental approach but with a reduced filling volume of 10 ml. Again, the OTR_max_ of a normal 250 ml shake flask is shown, which has a value of about 47 mmol/L/h. Also, in this case, an exponential growth can be seen in the perforated ring flask, which causes the OTR to rise significantly above the value of 47 mmol/L/h. After 11 h, the maximum of the curve is reached with an OTR of 120 mmol/L/h. In this case, there is no bending of the curve towards a plateau that would indicate an oxygen limitation. This is also in line with the data from the sulfite oxidation system (Figure 4), which shows an OTR of 118 mmol/L/h under these operating conditions. Since, for the mentioned reasons, a higher maximum oxygen transfer capacity of the medium can also be assumed here, an oxygen limitation in the cultivation can be excluded. After 11 h, there is a steep drop in the OTR curve, which suggests the depletion of glucose. Between 12 and 14 h, a small shoulder can be seen in the OTR curve. This may also be due to the metabolism of acetate that has been formed because of overflow metabolism. Indeed, it is well described in the literature that overflow metabolism in *E. coli* not only occurs under strict oxygen limitations but also at low levels of dissolved oxygen in the broth (Guest, 1992; Unden et al., 1994; Tseng et al., 1996; Cotter et al., 1997; Phue and Shiloach, 2005). Obviously, the avoidance of an oxygen limitation leads to a clear reduction of metabolic byproducts which are connected to overflow metabolism.

It is remarkable that at only 250 rpm an OTR can be achieved which can be compared with values normally only found in stirred tank reactors. Up to our best knowledge, comparable values have not yet been measured in normal 250 ml shake flasks. An extrapolation of the correlation of Meier et al. (2016) shows that the shaking frequency would have to be more than doubled to 590 rpm to achieve an OTR of 120 mmol/L/h. This is considered impractical because commercially available shakers hardly allow shaking frequencies higher than 300 rpm with a shaking diameter of d_0_ = 50 mm.

Figure 5C shows the results of another cultivation experiment with the same biological system as in Figures 5A,B. Here, however, a filling volume of V_L_ = 10 ml and the maximum possible shaking frequency of the applied shaker of *n* = 300 rpm were set. It should be mentioned that the curve has been shifted by 1.5 h in the diagram due to a shorter lag-phase of the growth curve compared to the other two curves as this experiment was not done in parallel to the other two cultivations and the preculture has been assumed to be more vital. Therefore, the shift has been done to allow a better optical comparison of the curves. Again, the OTR_max_ of the normal 250 ml shake flask is shown under these conditions being 57 mmol/L/h. Also, in this case, there is an exponential course of the OTR curve of the perforated ring flasks well beyond this value. The curve reaches its peak at 138 mmol/L/h before a steep drop occurs, which should be, again, due to the depletion of glucose. The consistently exponential shape of the OTR curve up to its maximum indicates the absence of an oxygen limitation. The data from the sulfite oxidation system (Figure 4) confirm this assumption. There a value of 144 mmol/L/ was achieved. As already described, the OTR_max_ in the medium is higher than in the sulfite oxidation system. This means that, although OTRs as usually only seen in stirred tank bioreactors are achieved, an oxygen limitation could be clearly prevented. In the further course of the curve, in contrast to the other two approaches, no diauxic peak can be seen after the steep drop of the OTR. This suggests that not only an oxygen limitation could be prevented but also the dissolved oxygen tension (DOT) in the medium can be kept high enough throughout the entire cultivation that even overflow metabolism can be avoided. Becker et al. (1996) found downregulation of tricarboxylic acid cycle key enzyme succinate dehydrogenase in batch cultures of *E. coli* K12 when the DOT fell below 10%. Simultaneously, the expression of formate dehydrogenase—as part of the anaerobic respiration system—was upregulated. As the OTR_max_ measured in the sulfite oxidation system is already higher than the highest measured OTR of 138 mmol/L/h in the cultivation and as the cultivation at *n* = 250 rpm and with V_L_ = 20 ml, (Figure 5A) shows an approximately 19% higher OTR_max_ than in the sulfite oxidation system it can reasonably be assumed that the DOT levels in the cultivation at *n* = 300 rpm and with V_L_ = 10 ml (Figure 5C) are indeed clearly above this critical DOT level of 10% throughout the whole cultivation. This would explain the absence of a diauxic peak in the OTR curve. A more quantitative confirmation by directly measuring the DOT levels during the cultivation was not done as the focus of this study is on the improvement of the gas-liquid mass transfer by the newly proposed flask design. A deeper quantitative characterization with respect to the DOT levels would need a carefully designed experimental set-up, e.g., by using oxygen-sensitive nanoparticles (Ladner et al., 2019) as conventional approaches to measure DOT in shake flasks can lead to erroneous results (Hansen et al., 2011). However, it could clearly be demonstrated that with the newly proposed perforated ring flasks design non-oxygen limited conditions at the OTR levels usually only seen in stirred tank bioreactors can be achieved in shaken bioreactors. Moreover, these high OTR levels can be achieved under practically relevant conditions with realistic filling volumes and shaking frequencies that can be applied to the most commercially available shakers.

In addition to the presented very high OTRs that could be achieved during the cultivations in the perforated ring flasks, the visual observation of the experiments is noteworthy. No splashing of the liquid could be observed. This means that at least some of the severe disadvantages that would come along with baffling can be prevented with the newly presented flask design.

## Conclusion

The newly introduced perforated ring flask design principle yields substantially higher gas-liquid mass transfer rates than conventional unbaffled shaken bioreactors under identical cultivation conditions. Yet, OTRs as elsewise only common in stirred tank bioreactors can be realized under practical operating conditions in most commercially available shakers. In fact, improvements in the gas-liquid mass transfer of over 200% and OTRs of up to 138 mmol/L/h in biological cultivation could be realized. Bigger ring diameters corresponded to higher OTR_max_ whereas the number, sizes, and geometries of the perforation showed no sensitivity regarding this parameter within the evaluated ranges. This strongly indicates that the increase in the gas-liquid mass transfer is mainly influenced by the capability to increase the total liquid film area *via* an increased surface area. In contrast to baffled shake flasks the increase in the gas-liquid mass transfer can be achieved while featuring much smoother hydrodynamics and without chaotic splashing of the liquid. An overview of the measured OTR values of the final flask design in comparison to data from the shake flask is given in Table 1.

**TABLE 1:** Maximum oxygen transfer capacities of the perforated ring flask with inner vessel diameter d_i_ = 80 mm, inner ring diameter d_ring,i_ = 70.6 mm, wall thickness d_wall_ = 2.2 mm, and 2 perforations of 3 mm × 3 mm size in comparison to a 250 ml shake flask.

	OTR_max_ (mmol/L/h)			
	Operating conditions	250 ml shake flask (Meier et al., 2016)	Ring flask	Improvement
Sulfite oxidation	V_L_ = 20 ml, *n* = 150 rpm	12.7	33.6	165%
	V_L_ = 20 ml, *n* = 200 rpm	17.2	52	202%
	V_L_ = 20 ml, *n* = 250 rpm	21.8	71.5	228%
	V_L_ = 20 ml, *n* = 300 rpm	26.5	88.6	234%
	V_L_ = 10 ml, *n* = 150 rpm	21.2	48.3	128%
	V_L_ = 10 ml, *n* = 200 rpm	28.8	84.1	192%
	V_L_ = 10 ml, *n* = 250 rpm	36.5	118	223%
	V_L_ = 10 ml, *n* = 300 rpm	44.3	144	225%
*E. coli* BL21 in Wilms-MOPS mineral medium	V_L_ = 20 ml, *n* = 250 rpm	28	85	204%
	V_L_ = 10 ml, *n* = 250 rpm	47	>120^a^	>155%
	V_L_ = 10 ml, *n* = 300 rpm	57	>138^a^	>142%

a No oxygen limitation detected. All experiments have been performed with a shaking diameter of d_0_ = 50 mm.

Consequently, this provides several new opportunities for cultivations in shaken bioreactors: for a good transferability of cultivation data from small-scale shaken bioreactors to stirred tank bioreactors and, hence, for reliable scale-ups the relevant parameters need to be mimicked in small-scale (Parekh et al., 2000; Funke et al., 2009). This also applies to the oxygen availability as one of the most important aspects of aerobic cultivations. The reliability of microbial screenings can be highly increased due to the avoidance of low dissolved oxygen levels or even oxygen limitations. Hence, the formation of unwanted metabolic byproducts, e.g., *via* overflow metabolism can be reduced or even prevented in many cases. Higher pH stabilities or even the opportunity to reduce buffer concentrations in the medium would be some of the benefits. Additionally, higher biomass concentrations could be achieved. All this would enable cultivation conditions much closer to production scale fermenters. Moreover, precultures and early seed train stages in production processes that are often not monitored can be cultivated with a low danger of oxygen limitations or negative baffling effects, thereby, contributing to higher overall process stability.

To achieve all this, it is neither necessary to invest in expensive equipment like special shakers that allow for unusual high shaking frequencies nor to establish elaborate setups for oxygen enrichment. Therefore, we believe that the new perforated ring flask principle has a high potential to considerably improve biotechnological screening and process development steps.

## Data Availability

The raw data supporting the conclusion of this article will be made available by the authors, without undue reservation.
