# Supplementary material for: Implementation of Perforated Concentric Ring Walls Considerably Improves Gas-Liquid Mass Transfer of Shaken Bioreactors
Source: Front Bioeng Biotechnol. 2022 May 12;10:894295. doi: 10.3389/fbioe.2022.894295 (PMC9135409; doi:10.3389/fbioe.2022.894295)
Supplement: Supplementary file 1 [file Presentation1.PPTX]

## Slide 1
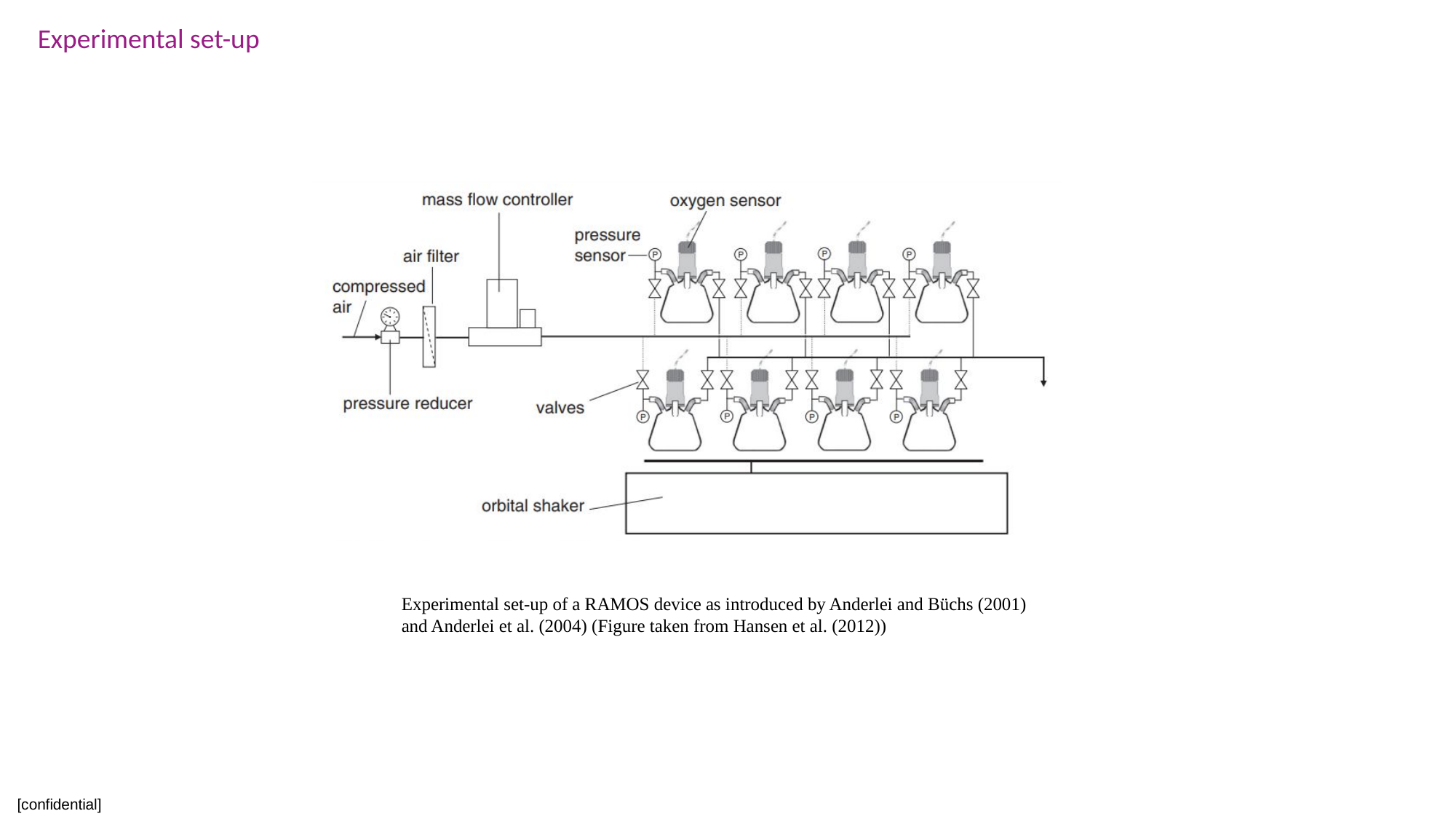

Experimental set-up
Experimental set-up of a RAMOS device as introduced by Anderlei and Büchs (2001) and Anderlei et al. (2004) (Figure taken from Hansen et al. (2012))

## Slide 2
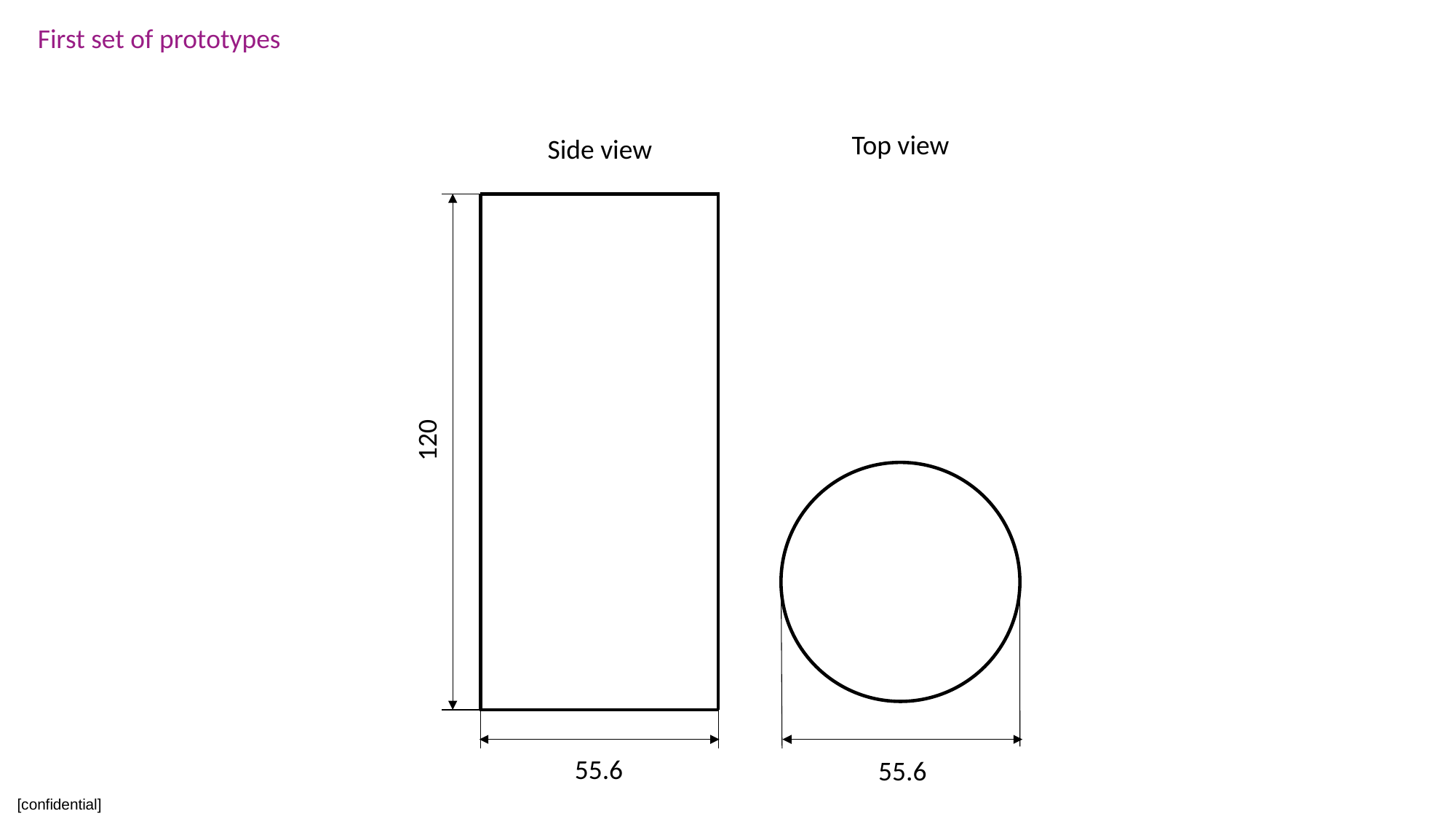

First set of prototypes
Top view
Side view
120
55.6
55.6

## Slide 3
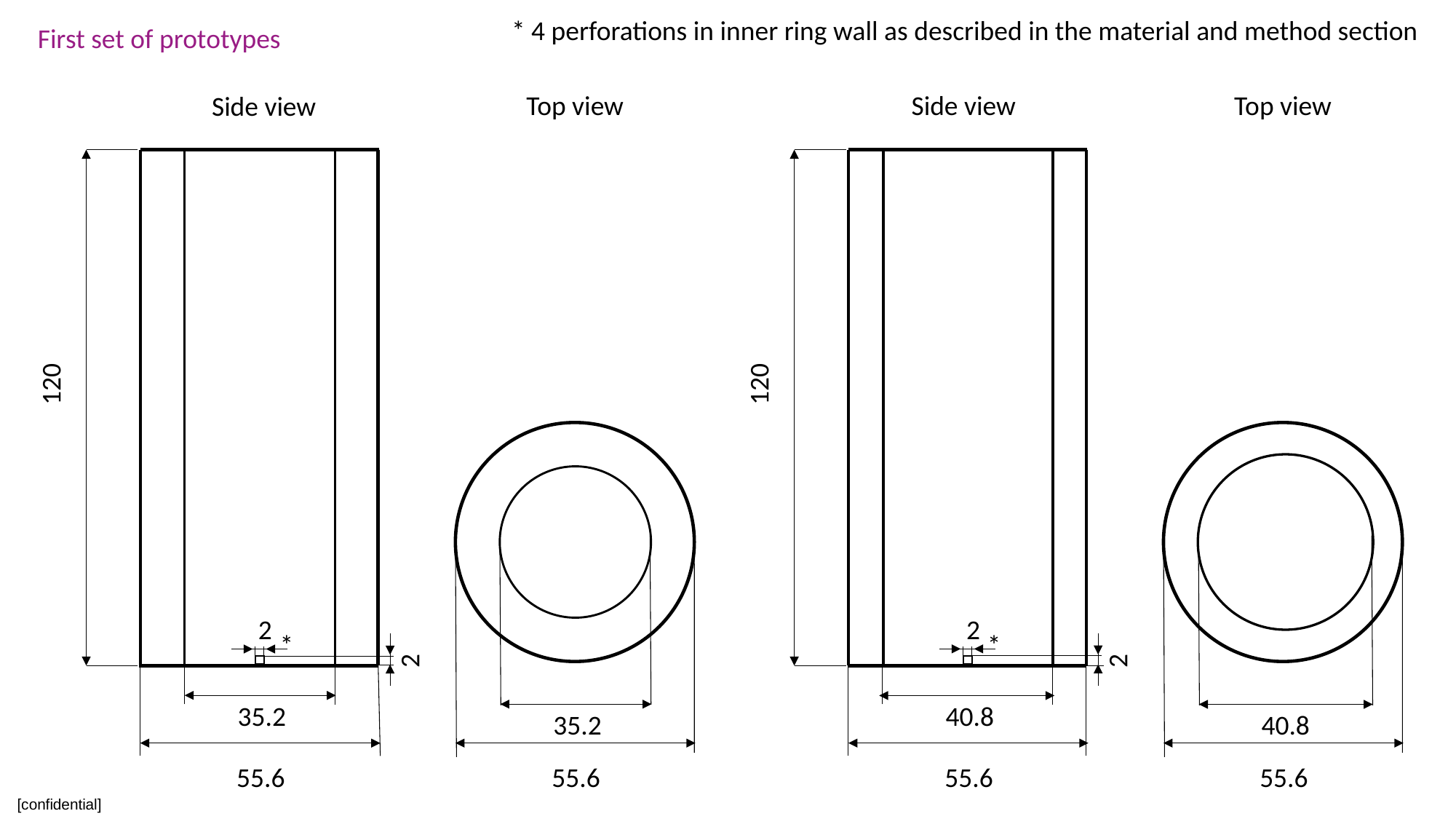

* 4 perforations in inner ring wall as described in the material and method section
First set of prototypes
Top view
Side view
Top view
Side view
120
120
2
2
*
*
2
2
40.8
35.2
40.8
35.2
55.6
55.6
55.6
55.6

## Slide 4
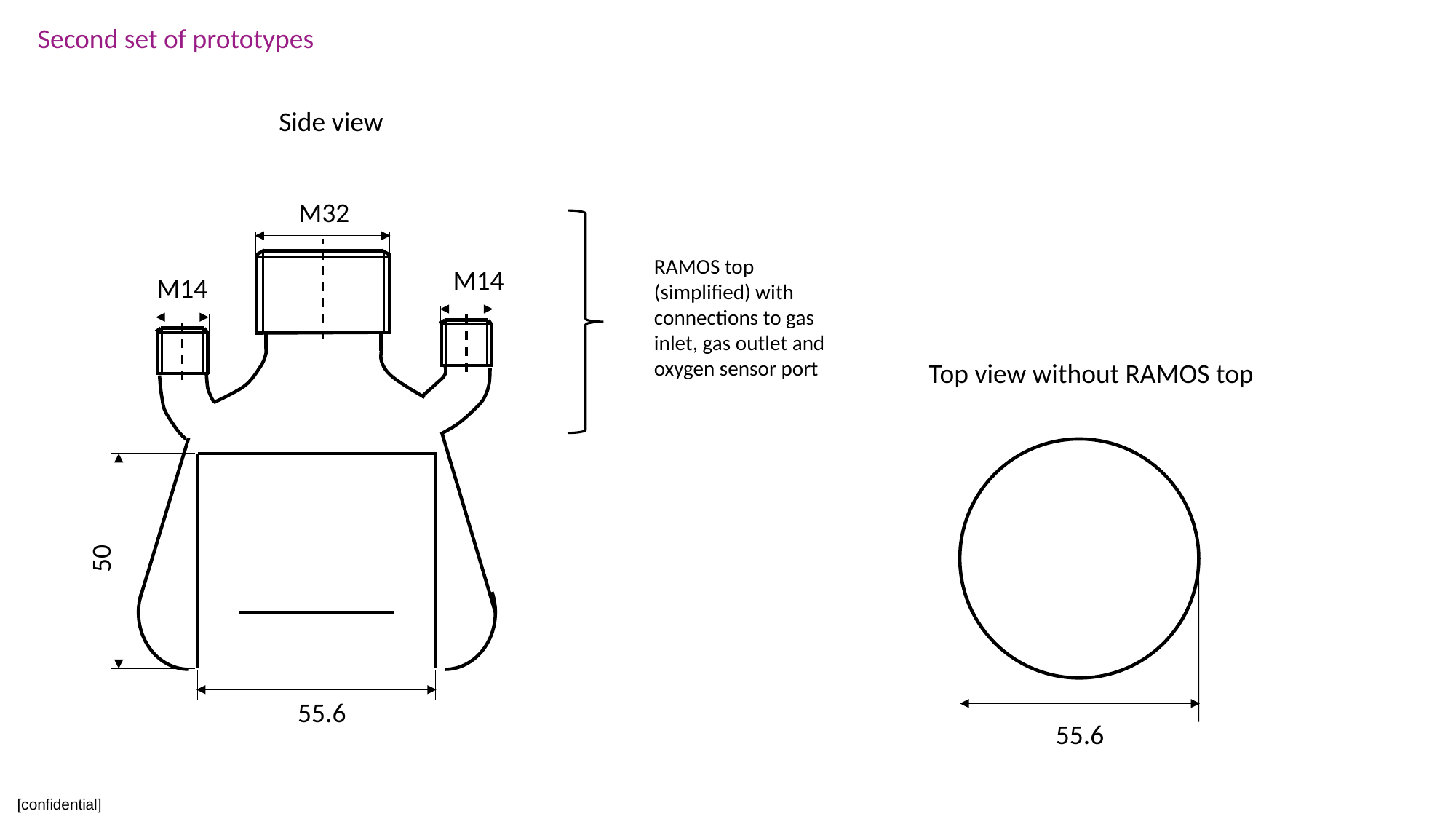

Second set of prototypes
Side view
M32
RAMOS top (simplified) with connections to gas inlet, gas outlet and oxygen sensor port
M14
M14
Top view without RAMOS top
50
55.6
55.6

## Slide 5
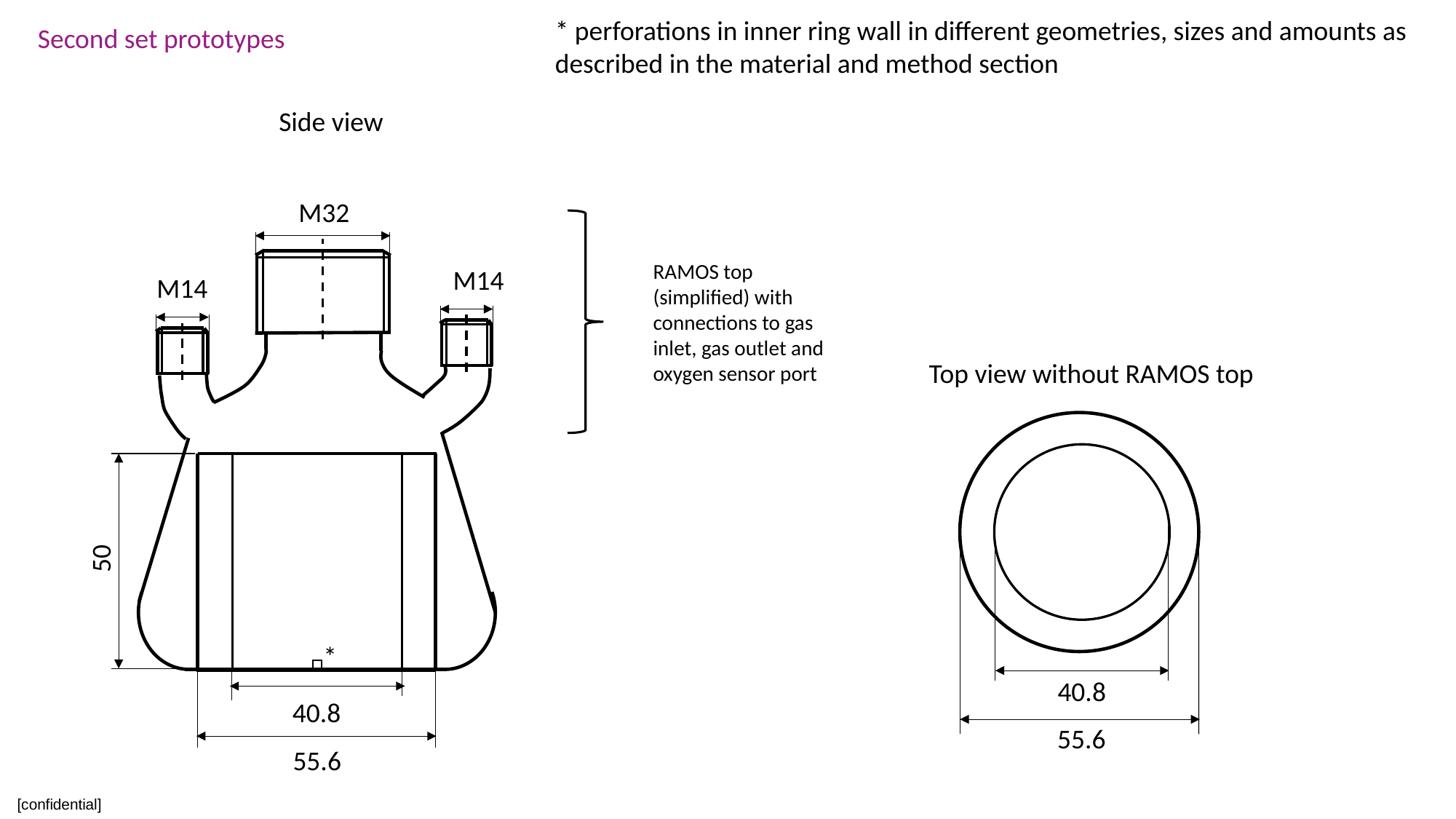

* perforations in inner ring wall in different geometries, sizes and amounts as described in the material and method section
Second set prototypes
Side view
M32
RAMOS top (simplified) with connections to gas inlet, gas outlet and oxygen sensor port
M14
M14
Top view without RAMOS top
50
*
40.8
40.8
55.6
55.6

## Slide 6
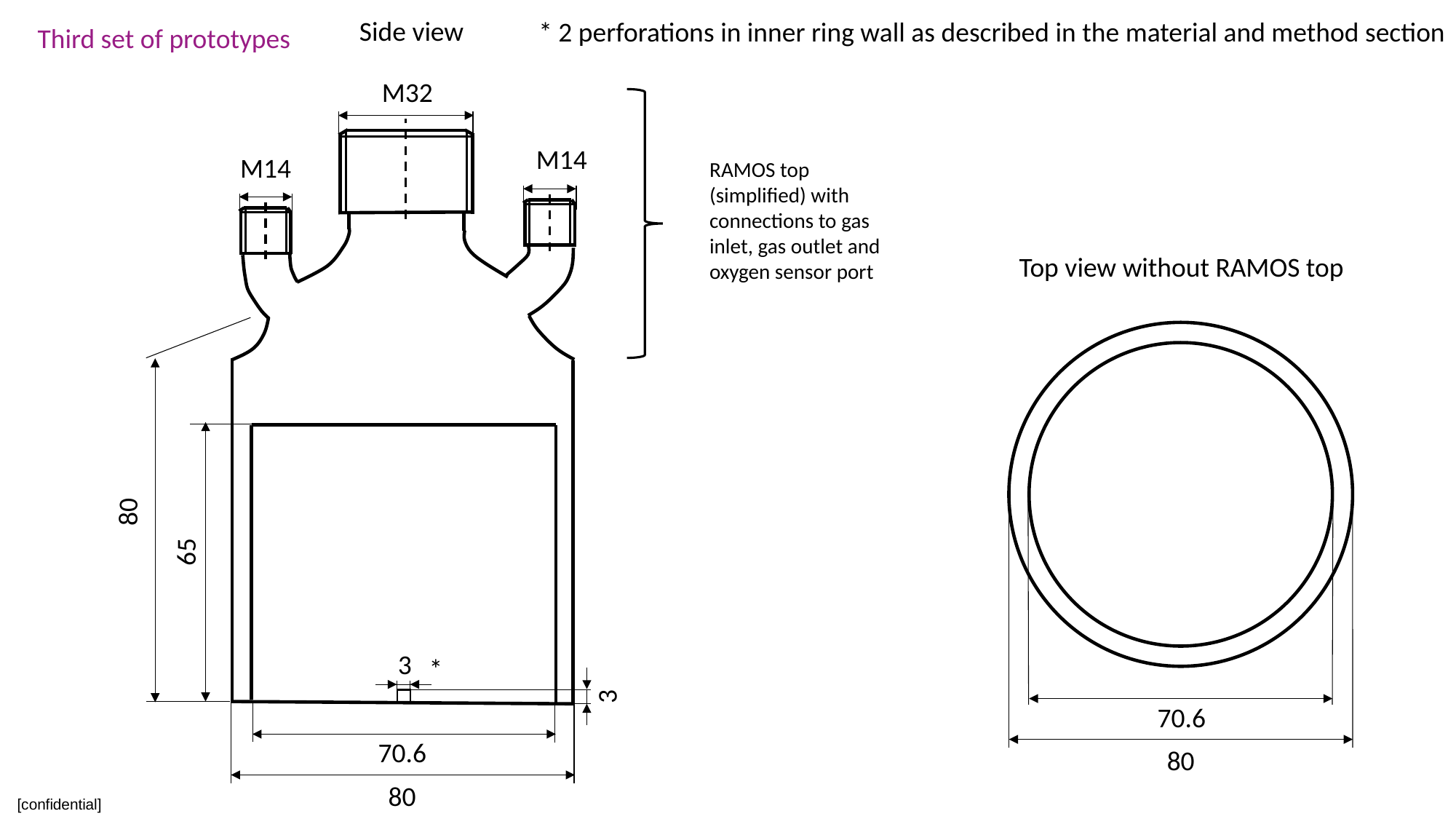

Side view
* 2 perforations in inner ring wall as described in the material and method section
Third set of prototypes
M32
M14
M14
RAMOS top (simplified) with connections to gas inlet, gas outlet and oxygen sensor port
Top view without RAMOS top
80
65
3
*
3
70.6
70.6
80
80
